# Knowledge, attitudes, and practices of COPD patients regarding acute exacerbations of COPD

**DOI:** 10.3389/fmed.2025.1620703

**Published:** 2025-09-03

**Authors:** Yuan Xia, Yahua Zhang, Dan Su

**Affiliations:** ^1^Pharmacy Department, Yuexi County Hospital, Anqing, China; ^2^Department of Respiratory Medicine, Yuexi County Hospital, Anqing, China; ^3^Department of Pharmacy, The First Affiliated Hospital of University of Science and Technology of China, Anhui Provincial Hospital, Hefei, China

**Keywords:** health literacy, patient education, structural equation modeling (SEM), self-management, cross-sectional study

## Abstract

**Objective:**

This study aims to assess the knowledge, attitudes, and practices (KAP) of patients with chronic obstructive pulmonary disease (COPD) concerning AECOPD.

**Methods:**

A cross-sectional survey was conducted at Yuexi County Hospital in Anhui Province, China, between April and December 2024. Data were collected using a structured questionnaire comprising demographic characteristics and KAP assessments. Adequate knowledge, positive attitudes, and proactive practices were defined as achieving at least 60% of the maximum possible score in each domain.

**Results:**

A total of 445 valid questionnaires were analyzed. Among the respondents, 332 (74.61%) were male, and 271 (60.90%) had been diagnosed with COPD for over a decade. The mean scores for knowledge, attitude, and practice were 7.25 ± 4.43 (range: 0–26), 36.91 ± 4.56 (range: 11–55), and 30.59 ± 3.76 (range: 10–50), respectively. Correlation analysis showed positive correlation between knowledge and attitude (r = 0.095, *p* = 0.045), knowledge and practice (r = 0.376, *p* < 0.001), and attitude and practice (r = 0.324, *p* < 0.001). SEM analysis demonstrated that knowledge directly influenced both attitude (*β* = 0.226, *p* = 0.036) and practice (β = 0.660, *p* = 0.008), while attitude also had a direct effect on practice (*β* = 0.299, *p* = 0.004). Additionally, knowledge exerted an indirect effect on practice through attitude (β = 0.067, *p* = 0.020).

**Conclusion:**

Despite generally positive attitudes and proactive practices, COPD patients exhibited insufficient knowledge about AECOPD, which may limit their ability to effectively manage exacerbations. Targeted education may enhance self-care and reduce exacerbation risk.

## Background

Chronic obstructive pulmonary disease (COPD) is a major global health burden, affecting approximately 213 million people and causing around 3.7 million deaths in 2021, according to the Global Burden of Disease 2021 (GBD 2021) estimates and the WHO 2024 fact sheet (1, 2). Acute exacerbation of chronic obstructive pulmonary disease (AECOPD) is a clinical state characterized by a rapid worsening of airway function and respiratory symptoms (3). It manifests as increased breathlessness, cough, sputum production, and sputum purulence, often requiring additional treatment (4). AECOPD are associated with poor prognosis, with hospitalization-related mortality rates reaching 15–30% within one year and up to 43% within two years, imposing a substantial financial burden on healthcare systems (5).

The most common triggers for AECOPD are respiratory infections (viral and bacterial), which account for approximately 70–80% of exacerbations (6). Other contributing factors include air pollution, non-adherence to maintenance therapy, and comorbidities. Studies in Chinese populations have reported a high recurrence rate of AECOPD, with approximately 20% of patients requiring re-hospitalization within 30 days and up to 60% experiencing at least one exacerbation within a year after their first event (7). A systematic review and meta-analysis reported AECOPD readmission rates of 11, 17, 17, 30, and 37% at 30, 60, 90, 180, and 365 days post-discharge, respectively (8). Self-management plays a crucial role in preventing AECOPD onset and progression, as early recognition of symptoms and prompt action can reduce hospitalization rates by up to 40% and significantly improve quality of life (7).

The Knowledge, Attitude, and Practice (KAP) framework provides a structured methodology for understanding patient behaviors and implementing targeted interventions. According to KAP theory, behavior change follows a sequential process: knowledge acquisition leads to attitude formation, which ultimately results in practice modification. This framework is particularly relevant as patient understanding and self-management practices significantly influence exacerbation outcomes (9). Investigating COPD patients’ KAP regarding AECOPD is crucial for identifying gaps in disease understanding, optimizing self-management strategies, and ultimately reducing exacerbation-related morbidity and healthcare burden.

Current KAP research on COPD patients has primarily focused on general disease knowledge (10), inhaler technique (11), smoking cessation (12), and pulmonary rehabilitation (13). Several studies have investigated patients’ overall understanding of COPD, but few have specifically examined patients’ knowledge, attitudes, and practices regarding AECOPD management. Therefore, this study aims to comprehensively assess the knowledge, attitudes, and practices regarding AECOPD among Chinese COPD patients.

## Methods

### Study design and participants

This cross-sectional study was conducted at Yuexi County Hospital in Anhui Province, China, between April and December 2024, focusing on hospitalized patients diagnosed with COPD. Ethical approval was granted by the Institutional Ethics Committee (Approval No.: 2024027), and informed consent was obtained from all participants prior to their inclusion in the study. Patients were eligible for participation if they met the following inclusion criteria: (1) a confirmed diagnosis of COPD (14), (2) aged 18 years or older, and (3) the ability to comprehend the questionnaire content and willingness to participate in the survey. No specific exclusion criteria were applied in this study.

### Questionnaire introduction

The questionnaire was developed based on established guidelines, including the Standardization of Clinical Assessment, Management and Follow-Up of Acute Hospitalized Exacerbation of COPD: A Europe-Wide Consensus (15), Pharmacologic Management of COPD Exacerbations: A Clinical Practice Guideline from the AAFP (16), and the Chinese Expert Consensus on Identifying and Managing High-Risk Patients with AECOPD (17), as well as relevant published literature (18, 19). Following the initial draft, the questionnaire underwent expert review and was refined based on feedback from two specialists in the field, thus enhancing the content validity. A pilot study involving 28 participants was conducted to assess its clarity and feasibility. The final version demonstrated good internal consistency, with an overall Cronbach’s *α* coefficient of 0.844. Additionally, participants in the pilot study were invited to identify any unclear or confusing items, and no concerns were raised, supporting the face validity of the questionnaire.

The finalized questionnaire, administered in Chinese, comprised four sections: demographic characteristics (including age, gender, education level, occupation, marital status, residence, monthly income, BMI, smoking status, duration of COPD, number of acute exacerbations in the past year, and severity of dyspnea), knowledge assessment, attitude evaluation, and practice assessment. The Modified Medical Research Council (mMRC) Dyspnea Scale was used to evaluated the severity of dyspnea in COPD patients (20). The knowledge section consisted of 13 items, each scored as “well understood” (2 points), “heard of” (1 point), or “unclear” (0 points), yielding a total score range of 0–26. The attitude dimension included 11 items evaluated on a five-point Likert scale, ranging from “strongly agree” (5 points) to “strongly disagree” (1 point), with possible scores ranging from 11 to 55. The practice section comprised 10 items, rated from “never” (1 point) to “always” (5 points), with a total score range of 10–50. Thresholds for adequate knowledge, positive attitudes, and proactive practices were set at ≥60.0% of the possible maximum score. This threshold was selected in accordance with previous KAP studies (21, 22), which commonly use 60% as a practical benchmark to indicate acceptable understanding or behavior levels in public health contexts.

### Questionnaire distribution and quality control

Participants were recruited through convenience sampling. The online questionnaire was created via the Wenjuanxing platform^1^ and a QR code for the survey was generated, which was distributed in hospital wards. These QR codes were prominently displayed in patient wards and at nurses’ stations to facilitate accessibility. COPD patients with a confirmed clinical diagnosis were instructed to scan the QR code and complete the survey using the Questionnaire Star platform. For elderly participants or those with difficulties in using electronic devices, family members were permitted to assist in questionnaire completion. Additionally, trained researchers provided support by reading the questions aloud when necessary, ensuring comprehension while refraining from offering any form of guidance or interpretation. After collecting the questionnaires, data quality checks were conducted. Questionnaires that took less than 2 min to complete, contained logical errors, or showed repeated pattern choices were deemed invalid and excluded. To ensure the validity and reliability of data by enhancing the seriousness of respondents’ answers, a common-sense trap question was included to identify and exclude inattentive respondents: Among the 56 ethnic groups in China, the Han ethnic group has the smallest population. Questionnaires with incorrect answers to this question were excluded as invalid questionnaires.

### Sample size

The required sample size for this study was determined using the formula for cross-sectional studies (23):

$$n=(Z^{2}\times P\times (1-P))/d^{2}$$

where *n* represents the required sample size, Z is the standard normal deviate (set at 1.96 for a 95% confidence level), P is the estimated prevalence of adequate knowledge, positive attitudes, or proactive practices (assumed to be 50% due to the lack of prior data to maximize sample size), and d is the margin of error (set at 5%). Substituting these values into the formula: *n* = (1.96^2^ × 0.5 × (1−0.5))/(0.05^2^) = 384.16. To account for potential non-responses, a 20% increase was applied, resulting in a final target sample size of 480 participants.

### Statistical analysis

Data analysis was performed using SPSS 27.0 (IBM, Armonk, NY, United States) and Amos 26.0 (IBM, Armonk, NY, United States). Continuous variables are presented as means with standard deviations (SD), while categorical variables are reported as frequencies and percentages (*n*, %). Normality was assessed using the Shapiro–Wilk test. For normally distributed continuous variables, comparisons between groups were conducted using the independent sample t-test or one-way analysis of variance (ANOVA). For non-normally distributed continuous variables, the Wilcoxon Mann–Whitney U test and Kruskal-Wallis test was applied. Correlation analysis was conducted using Spearman’s rank correlation coefficient to examine associations between KAP scores. A cutoff value of 60% of the maximum possible in each domain was considered indicative of adequate knowledge, positive attitudes, and proactive practices. The univariable analysis and multivariate logistic regression analysis was performed to identify independent predictors of adequate knowledge, positive attitudes, and proactive practices. Variables with *p* < 0.05 in the univariable analysis were included in the multivariable model. No multicollinearity was found based on variance inflation factors. Structural equation modeling (SEM) was utilized to explore the direct and indirect relationships among knowledge, attitude, and practice. The SEM analysis was conducted based on the following three assumptions: (H1) knowledge directly affects attitude, (H2) knowledge directly affects practice, and (H3) knowledge indirectly affects practice through attitude. Modification indices were examined, but no changes were made. Model fit were used should meet acceptable thresholds to ensure the model’s adequacy in explaining the observed relationships, including Root Mean Square Error of Approximation (RMSEA), Standardized Root Mean Square Residual (SRMR), Incremental Fit Index (IFI), Tucker–Lewis Index (TLI), and Comparative Fit Index (CFI). A two-tailed *p*-value <0.05 was considered statistically significant.

## Results

### Demographic information on participants

Initially, a total of 502 questionnaires were collected. The following were excluded (1). 6 cases with incorrect answers to trap questions; (2). 1 case who was under 18 years of age; (3). 50 cases with logical errors, or repeated pattern choices. The remaining 445 questionnaires were included in the final analysis, with a validity rate of 88.65%. Among them, 332 (74.61%) were male, 176 (39.55%) were aged 70–79, 382 (85.84%) lived in rural areas, 267 (60.00%) had a monthly income per capita of 2000–5,000 Yuan, 251 (56.40%) were former smoker, 271 (60.90%) had suffered from COPD for more than 10 years, 204 (45.84%) had 1–2 acute exacerbations in the past year. The primary source of COPD-related information was educational materials from hospitals or healthcare professionals (99.10%), followed by communication with relatives, friends, or other patients (27.42%), social media platforms (10.11%), traditional media (1.12%), and other sources (4.72%).

The mean knowledge, attitude, and practice scores were 7.25 ± 4.43 (possible range: 0–26), 36.91 ± 4.56 (possible range: 11–55), and 30.59 ± 3.76 (possible range: 10–50), respectively. Differences in knowledge scores were more likely to be found among participants with different gender (*p* < 0.001), residence (*p* = 0.032), monthly income per capita (*p* = 0.022), smoking status (*p* = 0.001), grade in mMRC Dyspnea Scale (*p* < 0.001), duration of COPD (*p* = 0.030), and number of acute exacerbations in the past year (*p* < 0.001). Differences in attitude scores were more likely to be found among those with different residences (*p* < 0.001), monthly income per capita (*p* < 0.001), grade in mMRC Dyspnea Scale (*p* < 0.001), and duration of COPD (*p* < 0.001). Differences in practice scores were more likely to be found among those with different residence (*p* < 0.001), monthly income per capita (*p* < 0.001), smoking status (*p* < 0.001), and number of acute exacerbations in the past year (*p* = 0.043) (Tables 1, 2).

**Table 1:** Demographic characteristics and KAP scores.

Characteristics	*N* (%)	Knowledge, mean ± SD	*P*	Attitude, mean ± SD	*P*	Practice, mean ± SD	*P*
Total score	445 (100%)	7.25 ± 4.43		36.91 ± 4.56		30.59 ± 3.76	
Gender			**<0.001**		0.481		0.360
Male	332 (74.61%)	7.62 ± 4.40		37.05 ± 4.55		30.73 ± 3.81	
Female	113 (25.39%)	6.15 ± 4.35		36.53 ± 4.61		30.19 ± 3.62	
Age (years)			0.191		0.372		0.828
<60	46 (10.34%)	8.39 ± 5.05		37.89 ± 5.01		31.02 ± 4.14	
60–69	155 (34.83%)	7.61 ± 4.69		36.99 ± 4.38		30.44 ± 3.61	
70–79	176 (39.55%)	6.90 ± 4.00		36.86 ± 4.56		30.57 ± 3.70	
≥80	68(15.28%)	6.57 ± 4.33		36.24 ± 4.64		30.71 ± 4.06	
Residence			**0.032**		**<0.001**		**<0.001**
Rural	382 (85.84%)	7.04 ± 4.28		36.33 ± 4.32		30.13 ± 3.51	
Urban	63 (14.16%)	8.54 ± 5.14		40.48 ± 4.41		33.38 ± 4.06	
Marital status			0.860		0.624		0.280
Married	410 (92.13%)	7.24 ± 4.39		36.88 ± 4.44		30.65 ± 3.73	
Other	35 (7.87%)	7.31 ± 4.95		37.29 ± 5.90		29.89 ± 4.18	
Monthly income per capita (Yuan)			**0.022**		**<0.001**		**<0.001**
<2000	152 (34.16%)	6.79 ± 4.37		36.45 ± 4.82		29.99 ± 3.88	
2000–5,000	267 (60.00%)	7.29 ± 4.31		36.60 ± 4.06		30.48 ± 3.39	
>5,000	26 (5.84%)	9.54 ± 5.40		42.85 ± 3.92		35.27 ± 3.72	
BMI			0.298		0.613		0.188
Underweight	152 (34.16%)	7.82 ± 4.75		36.66 ± 4.53		30.88 ± 4.39	
Normal	267 (60.00%)	6.94 ± 4.21		36.96 ± 4.56		30.31 ± 3.55	
Overweight	26 (5.84%)	7.28 ± 4.52		37.17 ± 4.65		30.95 ± 3.30	
Smoking status			**0.001**		0.082		**<0.001**
Currently smoking	69 (15.51%)	6.96 ± 5.11		37.99 ± 5.00		27.96 ± 3.41	
Never smoked	125 (28.09%)	6.41 ± 4.34		37.06 ± 4.65		31.46 ± 3.60	
Former smoker (quit)	251 (56.40%)	7.75 ± 4.22		36.55 ± 4.36		30.88 ± 3.63	

Bold values indicate statistical significance (*p* < 0.05).

**Table 2:** Clinical characteristics and KAP scores.

Characteristics	*N* (%)	Knowledge, mean ± SD	*P*	Attitude, mean ± SD	*P*	Practice, mean ± SD	*P*
Total score	445 (100%)	7.25 ± 4.43		36.91 ± 4.56		30.59 ± 3.76	
mMRC dyspnea scale			**<0.001**		**<0.001**		0.090
Grade 0	43 (9.66%)	6.07 ± 4.38		40.33 ± 5.62		31.70 ± 4.76	
Grade 1	109 (24.49%)	5.91 ± 3.46		37.87 ± 4.45		30.48 ± 3.57	
Grade 2	145 (32.58%)	7.38 ± 4.36		36.64 ± 3.95		30.47 ± 3.83	
Grade 3	110 (24.72%)	7.52 ± 4.36		35.95 ± 4.14		30.08 ± 3.39	
Grade 4	38 (8.54%)	11.16 ± 5.11		34.13 ± 4.21		31.61 ± 3.58	
Duration of COPD			**0.030**		**<0.001**		0.130
Less than 3 years	45(10.11%)	7.44 ± 5.11		39.04 ± 3.99		31.47 ± 3.73	
3–10 years	129(28.99%)	6.57 ± 4.50		37.80 ± 4.60		30.36 ± 3.83	
More than 10 years	271(60.90%)	7.54 ± 4.26		36.14 ± 4.46		30.56 ± 3.73	
Number of acute exacerbations in the past year			**<0.001**		0.538		**0.043**
0 times	91(20.45%)	5.80 ± 3.61		37.47 ± 5.02		30.20 ± 3.69	
1–2 times	204(45.84%)	6.41 ± 4.06		36.87 ± 4.40		30.29 ± 3.57	
3 or more times	150(33.71%)	9.27 ± 4.68		36.64 ± 4.49		31.24 ± 4.00	

mMRC dyspnea scale, modified medical research council dyspnea scale. Bold values indicate statistical significance (*p* < 0.05).

### Knowledge, attitude, and practice

The distribution of knowledge dimensions showed that the three questions with the highest number of participants choosing the “Not sure” option were “Are you aware of the common tools used for monitoring and identifying acute exacerbations in COPD patients, such as the PRO diary card or EXACT score?” (K8) with 99.10%, “When taking medications for other illnesses, are you aware that some drugs may worsen coughing symptoms?” (K9) with 96.18%, and “Improper use of medications may trigger acute exacerbation of COPD” (K3.3) with 87.42%. Meanwhile, only 18.20% of the participants were very familiar with the correct use of inhalation devices (e.g., exhaling completely before inhalation to empty the lungs, deeply and steadily inhaling the medication, holding your breath for about 10 s after inhaling, and rinsing your mouth after use) (K6). When it comes to the necessity of medication under a doctor’s supervision during AECOPD and the possibility of hospitalization in severe cases (K7), only 5.62% of the participants were familiar with it. In general, the participants’ knowledge level was quite low, and there is still a lot of room for education and improvement (Supplementary Table S1).

Responses to the attitude dimension showed that 49.21% strongly agreed and 34.16% agreed that they worried that acute exacerbations will recur (A7) and 34.16% strongly agreed and 31.2% agreed that COPD severely affects their quality of life (A6). However, 19.33% strongly disagreed and 36.85 disagreed that they are confident that they can take the correct actions during an acute exacerbation of COPD (A9), 8.26% strongly disagreed that (A6), as well as 8.31% strongly disagreed and 39.33% disagreed that preventing AECOPD is very important (A3) (Supplementary Table S2).

Responses to the practice dimension showed that 96.63% are very unlikely to seek vaccination regularly to prevent respiratory infections (P5.5), 78.65% are very unlikely to visit the hospital for check-ups to monitor changes in their condition (P3), 67.64% are very unlikely to avoid allergens, smoke, and inhalation of chemical substances (P5.6) (Supplementary Table S3).

### Univariate and multivariate analysis of knowledge, attitude, and practice dimensions

Multivariate logistic regression showed that aged above 60 years (OR < 1, 95% CI: [0.02 ~ 0.92], *p* < 0.05) and grade 4 in mMRC Dyspnea Scale (OR = 12.50, 95% CI: [2.22 ~ 70.21], *p* = 0.004) were independently associated with knowledge (Table 3). Meanwhile, living in urban (OR = 10.36, 95% CI: [2.41 ~ 44.52], *p* = 0.002), grade 3 in mMRC Dyspnea Scale (OR = 0.27, 95% CI: [0.07 ~ 0.95], *p* = 0.041), grade 4 in mMRC Dyspnea Scale (OR = 0.10, 95% CI: [0.03 ~ 0.42], *p* = 0.001), and suffering from COPD for more than 10 years (OR = 0.24, 95% CI: [0.07 ~ 0.85], *p* = 0.027) were independently associated with attitude (Table 4). Furthermore, knowledge score (OR = 1.17, 95% CI: [1.10 ~ 1.24], *p* < 0.001), attitude score (OR = 1.10, 95% CI: [1.04 ~ 1.16], *p* = 0.001), lived in urban (OR = 3.27, 95% CI: [1.55 ~ 6.90], *p* = 0.002), A monthly income per capita of more than 5,000 Yuan (OR = 4.90, 95% CI: [1.10 ~ 21.75], *p* = 0.037), having never smoked (OR = 9.89, 95% CI: [4.38 ~ 22.31], *p* < 0.001), and being a former smoker (OR = 5.02, 95% CI: [2.37 ~ 10.66], *p* < 0.001) were independently associated with proactive practice (Table 5).

**Table 3:** Univariate and multivariate logistics regression analysis for adequate knowledge.

Characteristics	Univariate logistic regression		Multivariate logistic regression	
	OR (95%CI)	*P*	OR (95%CI)	*P*
Gender				
Male	Ref.			
Female	0.59 (0.24 ~ 1.45)	0.248		
Age (years)				
<60	Ref.		Ref.	
60–69	0.44 (0.18 ~ 1.09)	0.075	0.32 (0.11 ~ 0.92)	0.034
70–79	0.20 (0.07 ~ 0.54)	0.002	0.16 (0.05 ~ 0.49)	0.002
≥80	0.19 (0.05 ~ 0.74)	0.017	0.08 (0.02 ~ 0.39)	0.002
Residence				
Rural	Ref.			
Urban	1.58 (0.66 ~ 3.79)	0.305		
Marital status				
Married	Ref.			
Other	1.11 (0.32 ~ 3.82)	0.872		
Monthly income per capita (Yuan)				
<2000	Ref.			
2000–5,000	1.04 (0.48 ~ 2.23)	0.924		
>5,000	2.33 (0.68 ~ 7.97)	0.177		
BMI				
Underweight	Ref.			
Normal	0.50 (0.23 ~ 1.08)	0.078		
Overweight	0.73 (0.28 ~ 1.89)	0.518		
Smoking status				
Currently smoking	Ref.			
Never smoked	0.45 (0.14 ~ 1.39)	0.163		
Former smoker (quit)	0.85 (0.35 ~ 2.08)	0.724		
mMRC dyspnea scale				
Grade 0	Ref.		Ref.	
Grade 1	0.00 (0.00 ~ Inf)	0.987	0.00 (0.00 ~ Inf)	0.987
Grade 2	1.52 (0.32 ~ 7.21)	0.599	2.16 (0.42 ~ 11.14)	0.357
Grade 3	2.28 (0.48 ~ 10.73)	0.298	2.61 (0.51 ~ 13.42)	0.250
Grade 4	9.46 (1.96 ~ 45.73)	0.005	12.50 (2.22 ~ 70.21)	0.004
Duration of COPD				
Less than 3 years	Ref.			
3–10 years	0.53 (0.16 ~ 1.71)	0.287		
More than 10 years	0.71 (0.25 ~ 1.97)	0.508		
Number of acute exacerbations in the past year				
0 times	Ref.		Ref.	
1–2 times	1.35 (0.36 ~ 5.12)	0.655	1.02 (0.24 ~ 4.27)	0.976
3 or more times	5.31 (1.55 ~ 18.24)	0.008	2.49 (0.64 ~ 9.73)	0.188

**Table 4:** Univariate and multivariate logistics regression analysis for positive attitude.

Characteristics	Univariate logistic regression		Multivariate logistic regression	
	OR (95%CI)	*P*	OR (95%CI)	*P*
Knowledge score	1.02 (0.97 ~ 1.07)	0.528		
Gender				
Male	Ref.			
Female	0.87 (0.52 ~ 1.44)	0.579		
Age (years)				
<60	Ref.			
60–69	0.81 (0.34 ~ 1.90)	0.628		
70–79	0.85 (0.36 ~ 1.98)	0.703		
≥80	0.41 (0.17 ~ 1.03)	0.057		
Residence				
Rural	Ref.		Ref.	
Urban	10.24 (2.46 ~ 42.66)	0.001	10.36 (2.41 ~ 44.52)	0.002
Marital status				
Married	Ref.			
Other	0.80 (0.36 ~ 1.77)	0.584		
Monthly income per capita (Yuan)				
<2000	Ref.			
2000–5,000	1.22 (0.76 ~ 1.94)	0.408		
>5,000	14683607.80 (0.00 ~ Inf)	0.983		
BMI				
Underweight	Ref.			
Normal	1.15 (0.69 ~ 1.93)	0.590		
Overweight	1.23 (0.63 ~ 2.40)	0.553		
Smoking status				
Currently smoking	Ref.			
Never smoked	0.84 (0.39 ~ 1.80)	0.658		
Former smoker (quit)	0.66 (0.33 ~ 1.30)	0.228		
mMRC dyspnea scale				
Grade 0	Ref.		Ref.	
Grade 1	0.41 (0.11 ~ 1.46)	0.168	0.49 (0.13 ~ 1.81)	0.283
Grade 2	0.33 (0.09 ~ 1.14)	0.079	0.45 (0.13 ~ 1.62)	0.222
Grade 3	0.18 (0.05 ~ 0.61)	0.006	0.27 (0.07 ~ 0.95)	0.041
Grade 4	0.08 (0.02 ~ 0.32)	<0.001	0.10 (0.03 ~ 0.42)	0.001
Duration of COPD				
Less than 3 years	Ref.		Ref.	
3–10 years	0.37 (0.10 ~ 1.30)	0.120	0.41 (0.11 ~ 1.50)	0.176
More than 10 years	0.19 (0.06 ~ 0.63)	0.007	0.24 (0.07 ~ 0.85)	0.027
Number of acute exacerbations in the past year				
0 times	REF.			
1–2 times	1.19 (0.64 ~ 2.21)	0.579		
3 or more times	0.68 (0.37 ~ 1.26)	0.219		

**Table 5:** Univariate and multivariate logistics regression analysis for proactive practice.

Characteristics	Univariate logistic regression		Multivariate logistic regression	
	OR (95%CI)	*P*	OR (95%CI)	*P*
Knowledge score	1.15 (1.10 ~ 1.20)	<0.001	1.17 (1.10 ~ 1.24)	<0.001
Attitude score	1.13 (1.08 ~ 1.18)	<0.001	1.10 (1.04 ~ 1.16)	0.001
Gender				
Male	Ref.			
Female	0.95 (0.62 ~ 1.46)	0.817		
Age (years)				
<60	Ref.			
60–69	0.74 (0.38 ~ 1.42)	0.361		
70–79	0.84 (0.44 ~ 1.60)	0.591		
≥80	0.64 (0.30 ~ 1.36)	0.249		
Residence				
Rural	Ref.			
Urban	5.07 (2.71 ~ 9.50)	<0.001	3.27 (1.55 ~ 6.90)	0.002
Marital status				
Married	Ref.			
Other	0.76 (0.38 ~ 1.54)	0.454		
Monthly income per capita (Yuan)				
<2000	Ref.		Ref.	
2000–5,000	1.29 (0.86 ~ 1.93)	0.217	1.08 (0.68 ~ 1.72)	0.748
>5,000	11.76 (3.38 ~ 40.88)	<0.001	4.90 (1.10 ~ 21.75)	0.037
BMI				
Underweight	Ref.			
Normal	0.89 (0.57 ~ 1.37)	0.593		
Overweight	1.30 (0.75 ~ 2.27)	0.351		
Smoking status				
Currently smoking	Ref.			
Never smoked	5.05 (2.58 ~ 9.91)	<0.001	9.89 (4.38 ~ 22.31)	<0.001
Former smoker (quit)	3.14 (1.69 ~ 5.86)	<0.001	5.02 (2.37 ~ 10.66)	<0.001
mMRC dyspnea scale				
Grade 0	Ref.		Ref.	
Grade 1	0.78 (0.38 ~ 1.58)	0.486	1.00 (0.43 ~ 2.32)	0.991
Grade 2	0.63 (0.32 ~ 1.24)	0.180	0.82 (0.35 ~ 1.91)	0.653
Grade 3	0.47 (0.23 ~ 0.96)	0.039	0.59 (0.24 ~ 1.44)	0.248
Grade 4	1.09 (0.45 ~ 2.63)	0.850	0.91 (0.30 ~ 2.78)	0.864
Duration of COPD				
Less than 3 years	Ref.			
3–10 years	0.63 (0.32 ~ 1.25)	0.190		
More than 10 years	0.66 (0.35 ~ 1.25)	0.208		
Number of acute exacerbations in the past year				
0 times	ref.			
1–2 times	0.82 (0.50 ~ 1.35)	0.435		
3 or more times	1.47 (0.87 ~ 2.48)	0.149		

### Correlation analysis of knowledge, attitude, and practice

Further correlation analysis revealed positive correlations between knowledge scores and attitude scores (r = 0.095, *p* = 0.045), as well as between knowledge scores and practice scores (r = 0.376, *p* < 0.001). Additionally, attitude scores were positively correlated with practice scores (r = 0.324, *p* < 0.001) (Supplementary Table S4).

### SEM analysis

**Figure 1:**
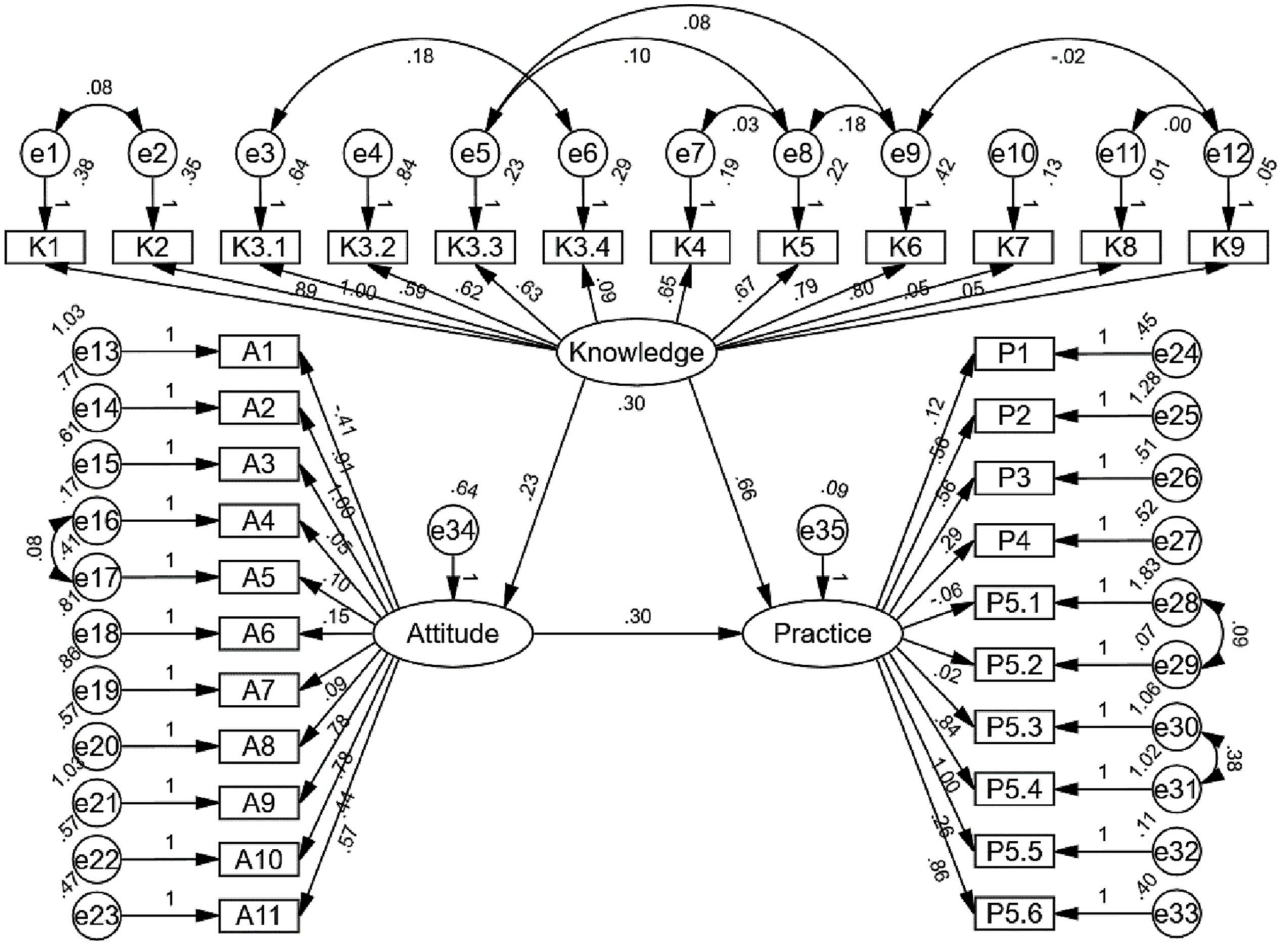
SEM analysis.

The SEM demonstrated a highly favorable model fit indices (CMIN/DF value: 2.778, RMSEA value: 0.063, IFI value: 0.758, TLI value: 0.730, and CFI value: 0.754), suggesting a well-fitting model (Supplementary Table S5). The results of SEM analysis showed that the direct effect of knowledge on both attitude (*β* = 0.226, *p* = 0.036) and practice (β = 0.660, *p* = 0.008), as well as of attitude on practice (β = 0.299, *p* = 0.004), furthermore, knowledge indirectly affected practice through attitude (β = 0.067, *p* = 0.020) (Table 6; Figure 1).

**Table 6:** Mediation analysis.

Model paths	Direct effects (95%CI)	*P*	Indirect effects (95%CI)	*P*
Knowledge → Attitude	0.226 (0.046, 0.444)	0.036	/	/
Knowledge → Practice	0.660 (0.508, 0.788)	0.008	0.067 (0.019, 0.153)	0.020
Attitude → Practice	0.299 (0.209, 0.483)	0.004	/	/

## Discussion

The results of this study indicated that patients with COPD exhibited inadequate knowledge, yet generally positive attitudes and proactive practices regarding AECOPD. The observed positive correlations among knowledge, attitudes, and practices suggest that enhancing knowledge may improve both attitudes and self-management practices. Therefore, targeted educational interventions should be implemented to increase COPD patients’ understanding of AECOPD, particularly among older individuals and lower dyspnea scales.

Our assessment of participants’ KAP scores revealed notable disparities across domains, with knowledge scores (7.25 ± 4.43, representing only 27.9% of the maximum possible score) being particularly low compared to attitude (36.91 ± 4.56, 67.1% of maximum) and practice (30.59 ± 3.76, 61.2% of maximum) scores. This pattern differs from findings in other KAP studies among COPD patients. A study conducted in urban China reported a higher knowledge rate (64.7%) but lower levels of proactive practices, while a Spanish survey identified similarly low knowledge with less favorable attitudes and poor self-management behaviors (9, 24). For instance, a study (9) reported higher knowledge levels (64.7% of maximum possible score) among urban Chinese COPD patients, while Hernandez et al. (24) found comparable knowledge deficits (33.2% of maximum) but lower practice scores (48.5% of maximum) in a Spanish cohort. This phenomenon of ‘practice-knowledge discordance’ has been observed in other chronic disease management studies (25), where patients develop functional management strategies through experience rather than formal education. The influence of social desirability bias may have contributed to the discrepancy between high reported attitudes and practices and low knowledge scores, as participants might have tended to give socially acceptable responses. However, this bias alone may not fully explain the pattern. Some participants may have formed habitual self-management behaviors through long-term interaction with healthcare professionals or repetitive exposure to community health messages, even in the absence of formal disease knowledge. The correlation analysis results demonstrated significant positive associations between knowledge, attitudes, and practices, indicating the interconnected nature of these domains in COPD management. Previous research has indicated that knowledge plays a fundamental role in shaping both attitudes and health-related behaviors (26, 27), yet many COPD patients in this study demonstrated a lack of awareness about fundamental aspects of their condition, particularly regarding triggers of exacerbations, appropriate medication use, and preventive strategies. Similar knowledge deficits have been reported in multiple COPD populations globally, including a study (28) found that Spanish COPD patients correctly answered only 35% of disease knowledge questions. SEM further revealed that knowledge not only directly influenced practice but also exerted an indirect effect through attitude, aligning with the theoretical framework of health behavior models that emphasize the cascading impact of knowledge on subsequent behavioral outcomes (29, 30).

The multivariate regression analysis identified several demographic and clinical factors significantly associated with knowledge, attitudes, and practices. Older patients exhibited significantly lower knowledge levels, a trend that has been widely reported in chronic disease populations, potentially due to cognitive decline, limited access to digital health resources, and lower health literacy (31, 32). The association between severe dyspnea and higher knowledge levels suggests that symptom burden may drive greater engagement with health information, as patients experiencing more distressing symptoms are more likely to seek medical advice or educational resources. However, the observed disparities between urban and rural populations indicate that access to structured educational programs remains uneven. Urban residents demonstrated significantly higher attitude and practice scores, a trend that has been documented in studies examining healthcare disparities, where urban populations benefit from greater healthcare accessibility, more frequent provider interactions, and higher exposure to public health campaigns (33, 34).

Our analysis of attitude scores and their determinants revealed complex patterns across different patient subgroups. While most patients expressed generally positive perspectives on COPD management, a significant proportion exhibited uncertainty about the effectiveness of preventive measures. The finding that patients with a longer disease duration were less likely to have a positive attitude suggests that sustained disease burden may lead to treatment fatigue and diminished motivation for long-term adherence, a phenomenon commonly observed in chronic illness management (35, 36). The association between symptom severity and less favorable attitudes may also reflect the psychological burden of COPD, as patients with more severe dyspnea often experience higher levels of anxiety and depression, which can negatively impact their perceptions of disease control and self-efficacy (37, 38). These results are in line with studies on other chronic respiratory diseases, where patients experiencing higher symptom burden often report greater frustration with disease management and reduced trust in treatment efficacy (39, 40).

The multivariate analysis of practice scores highlighted the significant impact of knowledge, attitudes, and sociodemographic factors of income and smoking status on patients’ self-management behaviors. The findings on practice further underscore the importance of knowledge and attitude in shaping self-management behaviors. Patients with higher knowledge and attitude scores were more likely to engage in proactive practices, supporting the widely established relationship between health literacy and adherence to treatment recommendations (41, 42). However, significant disparities were observed based on socioeconomic status and smoking history. Patients with higher income levels were more likely to engage in preventive behaviors, which aligns with existing literature demonstrating that financial security facilitates better access to healthcare resources, medications, and follow-up visits (43, 44). Meanwhile, the significantly lower practice scores among current smokers highlight the persistent challenge of smoking cessation in COPD management. Previous studies have indicated that current smokers are less likely to adhere to treatment regimens and engage in preventive behaviors, potentially due to lower risk perception and reduced motivation to modify lifestyle habits (45, 46).

Detailed examination of individual knowledge, attitude, and practice items in the Supplementary material revealed specific areas of concern that require targeted intervention. The Supplementary material further illustrate the extent of knowledge gaps among COPD patients. A particularly concerning finding was the lack of knowledge regarding proper inhaler technique and medication management during acute exacerbations. Only 18.20% of participants reported being “very familiar” with the correct use of inhalation devices, including crucial techniques such as complete exhalation before inhalation, proper inhalation technique, breath-holding, and mouth rinsing after use. Similarly, only 5.62% were familiar with the necessity of medication under a doctor’s supervision during acute exacerbations and the possibility of hospitalization in severe cases. These findings align with previous research highlighting widespread deficiencies in inhaler technique knowledge and practice among COPD patients. For instance, one study found that 79% of COPD patients made at least one error in inhaler technique, with critical errors that significantly reduced drug delivery occurring in 28% of patients (11). Similarly, a systematic review reported that 50–80% of patients demonstrated incorrect inhaler technique, with errors persisting even after repeated instruction (47, 48). This is particularly problematic given that proper inhaler technique is essential for effective drug delivery to the airways, with studies showing that incorrect technique can reduce the amount of medication reaching the lungs by up to 50%, leading to poor symptom control and increased exacerbation rates (48). Moreover, the low awareness about proper medication management during exacerbations is concerning, as timely and appropriate medication adjustments under medical supervision are crucial for preventing exacerbation progression and reducing hospitalization risk. Previous research has demonstrated that delayed treatment or inappropriate self-medication during exacerbations can lead to prolonged recovery time and increased healthcare utilization (15, 16). A large proportion of respondents were unable to correctly identify key aspects of disease management, including medication use, monitoring tools, and preventive strategies. This is concerning given that previous research has shown that patients who are unaware of proper medication techniques, such as inhaler use, often experience poorer symptom control and increased exacerbation rates (47, 48). The lack of awareness about monitoring tools is particularly notable, as self-monitoring has been linked to improved outcomes in COPD management by enabling early intervention during exacerbations (49, 50). Similarly, the attitude data suggest that while most patients acknowledge the impact of COPD on their quality of life, many remain unconvinced about the effectiveness of proactive disease management strategies. The practice data further support this concern, with particularly low adherence to regular medical check-ups and vaccinations, both of which are crucial in preventing severe exacerbations. Studies in comparable populations have suggested that vaccine hesitancy remains a major issue in COPD management, often influenced by misinformation, lack of provider recommendation, and accessibility barriers (51, 52).

Based on our findings of inadequate knowledge despite positive attitudes and practices, a multifaceted intervention strategy is needed to address these gaps. Given the strong association between knowledge and practice, expanding patient education programs should be prioritized. Traditional education sessions in clinical settings have been shown to be effective but may not reach all patients, particularly those in rural areas. Digital health interventions, including mobile-based education programs, telehealth consultations, and interactive learning modules, have demonstrated success in improving disease literacy and self-management in similar populations (53, 54). Healthcare providers should also adopt a more structured approach to patient education by integrating regular reinforcement of key COPD management principles into routine consultations. Studies have shown that repeated exposure to health information enhances retention and long-term behavioral change, particularly in chronic disease populations (55, 56). Cognitive-behavioral therapy and structured counseling programs have been effective in improving treatment adherence and self-efficacy in patients with other chronic illnesses and should be considered as part of COPD care (57). Moreover, targeted interventions should focus on populations at higher risk of poor disease management, including older adults and those with lower socioeconomic status.

This study has several limitations. First, as a cross-sectional survey, it only captures associations rather than causal relationships, limiting the ability to infer the impact of knowledge and attitudes on practices over time. Second, the data were collected from a single hospital, which may restrict the generalizability of the findings to broader COPD populations with different healthcare access and socioeconomic backgrounds. Third, self-reported measures of knowledge, attitudes, and practices are subject to response bias, as participants may have overestimated or underestimated their actual understanding and behaviors.

In conclusion, COPD patients demonstrated inadequate knowledge but exhibited positive attitudes and proactive practices regarding AECOPD. The observed correlations between knowledge, attitudes, and practices suggest that improving patient knowledge may enhance both attitudes and self-management behaviors. Targeted educational interventions focusing on COPD management, particularly for older patients and those with less severe symptoms, may be beneficial in improving disease awareness and promoting better self-care practices. For future implementation, mobile-based education tools and telehealth consultations could be explored as accessible platforms in low-resource settings. Ultimately reducing the risk of AECOPD. Additionally, the use of convenience sampling may limit representativeness. The 60% threshold for KAP classification was adapted from previous studies but remains arbitrary.

## Data Availability

The original contributions presented in the study are included in the article/Supplementary material, further inquiries can be directed to the corresponding author.

## Footnotes

^1^ https://www.wjx.cn/
