# Supplementary material for: Knowledge, attitudes, and practices of COPD patients regarding acute exacerbations of COPD
Source: Front Med (Lausanne). 2025 Sep 3;12:1620703. doi: 10.3389/fmed.2025.1620703 (PMC12440717; doi:10.3389/fmed.2025.1620703)
Supplement: Supplementary file 1 [file Table_1.docx]

**Supplementary table 1. Distribution of knowledge dimension responses**

| Items, n (%) | Very familiar | Heard of it | Not sure |
| --- | --- | --- | --- |
| 1. Chronic obstructive pulmonary disease (COPD) is a common chronic respiratory disease characterized by airflow limitation, leading to difficulty breathing. | 92 (20.67%) | 134 (30.11%) | 219 (49.21%) |
| 2.Acute exacerbation of COPD refers to a sudden worsening of COPD symptoms, typically including increased shortness of breath, worsened coughing, and increased sputum production, often requiring additional treatment. | 92 (20.67%) | 78 (17.53%) | 275 (61.80%) |
| 3. Are you aware of the following factors that may trigger acute exacerbation of COPD? |  |  |  |
| 3.1 Respiratory infections | 321 (72.13%) | 14 (3.15%) | 110 (24.72%) |
| 3.2 Smoking or air pollution | 228(51.24%) | 17(3.82%) | 200(44.94%) |
| 3.3 Improper use of medications | 40(8.99%) | 16(3.60%) | 389(87.42%) |
| 3.4 Temperature changes | 403(90.56%) | 8(1.80%) | 34(7.64%) |
| 4. AECOPD are often preventable. Measures such as quitting smoking, avoiding air pollution, regularly using maintenance respiratory medications, getting influenza and pneumococcal vaccines, and maintaining good personal hygiene can reduce the risk of future exacerbations. | 26 (5.84%) | 75 (16.85%) | 344 (77.30%) |
| 5. Inhalation therapy is the first-choice route for maintenance treatment of COPD. It maximizes symptom improvement, prevents disease progression, and minimizes adverse reactions. | 34 (7.64%) | 59 (13.26%) | 352 (79.10%) |
| 6. Are you familiar with the correct use of inhalation devices (e.g., exhaling completely before inhalation to empty the lungs, deeply and steadily inhaling the medication, holding your breath for about 10 seconds after inhaling, and rinsing your mouth after use)? | 81 (18.20%) | 32 (7.19%) | 332 (74.61%) |
| 7. During AECOPD, medications should be used under the guidance of a physician. This may include increasing inhaled bronchodilators, corticosteroids, and antibiotics. In severe cases, hospitalization may be required. | 25 (5.62%) | 86 (19.33%) | 334 (75.06%) |
| 8. Are you aware of the common tools used for monitoring and identifying acute exacerbations in COPD patients, such as the PRO diary card or EXACT score? | 0 | 4 (0.90%) | 441 (99.10%) |
| 9. When taking medications for other illnesses, are you aware that some drugs may worsen coughing symptoms? | 2 (0.45%) | 15 (3.37%) | 428 (96.18%) |

**Supplementary table 2. Distribution of attitude dimension responses**

| Items, n (%) | Strongly agree | Agree | Neutral | Disagree | Strongly disagree |
| --- | --- | --- | --- | --- | --- |
| 1. I believe COPD severely affects my quality of life. | 151 (34.16%) | 139 (31.24%) | 90 (20.22%) | 60 (13.48%) | 4 (0.90%) |
| 2. I believe regular check-ups and early detection of acute exacerbations are necessary. | 48 (10.79%) | 122 (27.42%) | 75 (16.85%) | 175 (39.33%) | 25 (5.62%) |
| 3. I believe preventing AECOPD is very important. | 46 (10.34%) | 75 (16.85%) | 112 (25.17%) | 175 (39.33%) | 37 (8.31%) |
| 4. I am willing to adjust my treatment plan based on my doctor’s recommendations. | 366 (82.25%) | 75 (16.85%) | 4 (0.90%) | 0 | 0 |
| 5. I am willing to adhere to daily medication. | 296 (66.52%) | 128 (28.76%) | 13 (2.92%) | 7 (1.57%) | 1 (0.22%) |
| 6. I am concerned that long-term medication use may cause adverse reactions or side effects. | 15 (3.37%) | 54 (12.13%) | 61 (13.71%) | 284 (63.82%) | 31 (6.97%) |
| 7. I am worried that acute exacerbations will recur. | 219 (49.21%) | 152 (34.16%) | 41 (9.21%) | 29 (6.52%) | 4 (0.90%) |
| 8. I believe proper daily management can reduce the risk of AECOPD. | 23 (5.17%) | 94 (21.12%) | 98 (22.02%) | 217 (48.76%) | 13 (2.92%) |
| 9. I am confident that I can take the correct actions during an acute exacerbation of COPD. | 24 (5.39%) | 105 (23.60%) | 66 (14.83%) | 164 (36.85%) | 86(19.33%) |
| 10. If I am unsure about how to take medication, I will actively consult healthcare professionals. | 9 (2.02%) | 51 (11.46%) | 28 (6.29%) | 325 (73.03%) | 32 (7.19%) |
| 11. I am willing to learn more about AECOPD. | 44(9.89%) | 218(48.99%) | 131(29.44%) | 51(11.46%) | 1(0.22%) |

**Supplementary table 3. Distribution of practice dimension responses**

| Items, n (%) | Extremely likely | Likely | Neutral | Unlikely | Extremely unlikely |
| --- | --- | --- | --- | --- | --- |
| 1. I will adhere to daily medication as prescribed by my doctor. | 44 (9.89%) | 218 (48.99%) | 131 (29.44%) | 51 (11.46%) | 1 (0.22%) |
| 2. I will use antibiotics on my own when experiencing cough symptoms. | 14 (3.15%) | 52 (11.69%) | 93 (20.90%) | 95 (21.35%) | 191(42.92%) |
| 3. I regularly visit the hospital for check-ups to monitor changes in my condition. | 1 (0.22%) | 15 (3.37%) | 33 (7.42%) | 46 (10.34%) | 350(78.65%) |
| 4. If I experience symptoms of an acute exacerbation of COPD, I will seek medical help promptly. | 226(50.79%) | 177 (39.78%) | 28 (6.29%) | 14 (3.15%) | 0 |
| 5. In daily life, I pay attention to the following |  |  |  |  |  |
| 5.1 Avoid smoking or exposure to secondhand smoke | 292 (65.62%) | 84 (18.88%) | 7 (1.57%) | 4 (0.90%) | 58(13.03%) |
| 5.2 Stay warm and prevent catching cold | 420(94.38%) | 22(4.94%) | 3(0.67%) | 0 | 0 |
| 5.3 Ensure adequate rest, engage in moderate physical activity, and exercise regularly | 63(14.16%) | 122(27.42%) | 97(21.80%) | 149(33.48%) | 14(3.15%) |
| 5.4 Avoid alcohol, maintain a healthy diet, and ensure balanced nutrition | 67(15.06%) | 136(30.56%) | 97(21.80%) | 123(27.64%) | 22(4.94%) |
| 5.5 Get vaccinated regularly (e.g., flu vaccine) to prevent respiratory infections | 2(0.45%) | 1(0.22%) | 2(0.45%) | 10(2.25%) | 430(96.63%) |
| 5.6 Avoid allergens, smoke, and inhalation of chemical substances | 1(0.22%) | 16(3.60%) | 30(6.74%) | 97(21.80%) | 301(67.64%) |

**Supplementary table 4. Correlation analysis**

|  | Knowledge | Attitude | Practice |
| --- | --- | --- | --- |
| Knowledge | 1 |  |  |
| Attitude | 0.095 (P=0.045) | 1 |  |
| Practice | 0.376 (P<0.001) | 0.324 (P<0.001) | 1 |

**Supplementary table 5. Model fit results**

|  | Ref. | Measured results |
| --- | --- | --- |
| CMIN/DF | 1-3 excellent，3-5 good | 2.778 |
| RMSEA |  | 0.063 |
| IFI | >0.8 good | 0.758 |
| TLI | >0.8 good | 0.730 |
| CFI | >0.8 good | 0.754 |
